# Persistence of Coastal Vegetation in Supratidal Zones of Northern China

**DOI:** 10.1371/journal.pone.0079964

**Published:** 2013-11-05

**Authors:** Hongxiao Yang, Jianmin Chu

**Affiliations:** 1 College of Resources and Environment, Qingdao Agricultural University, Chengyang, Qingdao, Shandong Province, China; 2 Key Laboratory of Tree Breeding and Cultivation, State Forestry Administration; Research Institute of Forestry, Chinese Academy of Forestry, Beijing, China; University of Waikato (National Institute of Water and Atmospheric Research), New Zealand

## Abstract

Coastal vegetation comprises a number of coastal specialists and terrestrial generalists. It remains unclear how they persist on disturbed and undisturbed coastal conditions. We tested the hypothesis that coastal specialists may be superior to terrestrial generalists on supratidal zones of coasts, but their superiority can be influenced by human disturbances. Eight separate sandy coasts of the Shandong Peninsula were sampled, representing for disturbed and undisturbed sandy coasts. Plants growing on their supratidal zones were surveyed. On this basis, we compared the relative dominances, niche widths, and commonness of all species, and also analyzed species diversities of the coasts. Coastal specialists were found to be more common and widespread on supratidal zones of the sandy coasts than terrestrial generalists haphazardly invading from hinterlands. Coastal specialists exhibited lower Sørensen dissimilarities than terrestrial generalists among the coasts. Tourist trampling seemed more detrimental than pond fishery to coastal vegetation. Relative to terrestrial generalists, coastal specialists responded to human disturbances more deterministically, with steady decreases in species diversities. These evidences verify that coastal specialists are intrinsically superior to terrestrial generalists on supratidal zones of coasts, especially of undisturbed coasts, because their dispersal among coasts adapts well to local storm surge regime. They also validate that human disturbances can depress the superiority of coastal specialists, partly by inducing invasion of terrestrial generalists.

## Introduction

Coastal vegetation protects coastlines from receding and other disasters [1–3]. Welfare of coastal residents is closely related with coastal vegetation [4–6]. Unfortunately, human activities such as fishery and tourism are endangering coastal plants, causing ecological degradation and species extinction somewhere [7–9]. Coastal vegetation must be managed scientifically and conserved timely [10,11]. This needs further understanding on relationships between coastal plants and habitats. The Shandong Peninsula, a typical coastal area of northern China, provides appropriate sites for such studies.

The Shandong Peninsula is the largest peninsula in China, partially surrounded by the Yellow and Bo Seas that are connected to the Northwestern Pacific Ocean. The peninsula margins present distinctive vegetation types, i.e., coastal vegetation [12–14]. As far as sandy coasts are concerned, coastal vegetation normally grows in supratidal zones of the coasts and is made up of a number of coastal specialists and terrestrial generalists. By nature, the coastal specialists are distributed exclusively on supratidal zones, whereas terrestrial generalists are distributed much broader, being quite usual or common in hinterlands. Researchers reported that coastal specialists, which are suitable for seawater dispersal, mostly bear buoyant seeds and tolerate seawater inundation as well, unlike most hinterland plants, which are usually suitable for wind or animal dispersal [15–17]. These traits may influence plant survivability or persistence on disturbed and undisturbed coasts. 

Coastal tourism and pond fisheries are currently booming in the peninsula, as in many coastal areas worldwide [7,8,18]. From June to October every year, numerous people crowd on sandy coasts near cities or towns for swimming or recreation. They inevitably trample on coastal vegetation. Although some sandy coasts near remote villages do not suffer tourist trampling, they may be exploited as ponds for cultivating fishes, shrimps or other aquatic animals. As a result, habitats of coastal plants are reduced and suffer from trampling, chipping and digging to some extent. These disturbances may have caused some deviations of coastal vegetation from original states. So far, it remains unclear how coastal specialists and terrestrial generalists respond to these disturbances. 

Supratidal zones, by their nature, are different from hinterlands for occasional inundation of storm surges [16,19,20]. Once storm surges take place, seawater may surge up beyond average high tide lines in form of violent waves, so as to wash supratidal zones and coastal vegetation temporarily. Coastal specialists may have evolved good adaptations to storm surges, but terrestrial generalists may not. Additionally, the prevailing disturbances of tourist trampling and pond fishery must have affected coastal vegetation somehow, but perhaps differently for coastal specialists and terrestrial generalists. In this study, we investigated and compared coastal vegetation along sandy coasts of the Shandong Peninsula. We attempted to test the hypothesis that coastal specialists are intrinsically superior to terrestrial generalists on supratidal zones because of some better adaptations, but their superiority is depressed or influenced by human disturbances. The central question we concerned is how coastal plants persist on disturbed and undisturbed supratidal zones of sandy coasts. 

## Materials and Methods

### Study sites

The Shandong Peninsula (35° - 38° N, 119.5° - 123° E) is surrounded incompletely by the Bo and Yellow Seas. Annual precipitation ranges from 650 to 850 mm. The monthly mean temperature varies between -3 °C (January) and 25 °C (August). Sandy, muddy and rocky coasts make up the coastlines. On sandy coasts, the intertidal flats are always free of terrestrial plants perhaps because they lack tolerances to semidiurnal tides. The supratidal zones can be inundated occasionally by storm surges, which take place when spring tides are further promoted by coincident typhoons or hurricanes [21]. On average, a storm surge occurs every three years and an extraordinary one occurs every 20 - 30 years [22]. A storm surge lasts several hours at a coast. Adapted to such inundation regime, distinctive vegetation, i.e. sandy coastal vegetation, has come into being along supratidal zones of the sandy coasts.

The original vegetation had ever covered throughout the supratidal zones of the sandy coasts, in widths of tens of meters landward behind average high tide lines. However, it is disturbed increasingly because of tourist trampling, pond fishery and other human activities. The vegetation consists of grasses, forbs, sedges and shrubs, and they are usually up to 50 - 60 cm tall. Some plants are specialists affiliated to sandy coasts, such as *Carex kobomugi*, *Calystegia soldanella*, *Leymus mollis*, *Vitex trifolia* var. *simplicifolia*, *Messerschmidia sibirica*, *Carex pumila*, and *Glehnia littoralis* [12,23,24]. Some are generalists considerably common in broad hinterlands, such as *Cynodon dactylon*, *Cyperus microiria*, *Lespedeza bicolor*, *Lespedeza davurica*, *Imperata cylindrical*, *Oenothera erythrosepala*, *Lepidium apetalum*. On muddy coasts, there grows other plants such as *Suaeda heteroptera*, *Suaeda salsa*, *Spartina alterniflora*, *Phragmites australis*, *Tamarix chinensis*. On rocky coasts, terrestrial plants include *Pinus thunbergii*, *Pinus densiflora*, *Robinia pseudoacacia*, *Lespedeza bicolor*, *Vitex negundo* var. *heterophylla*, *Themeda triandra*, *Gypsophila oldhaminana*, and so on [23,24]. 

### Field survey

**Figure 1:**
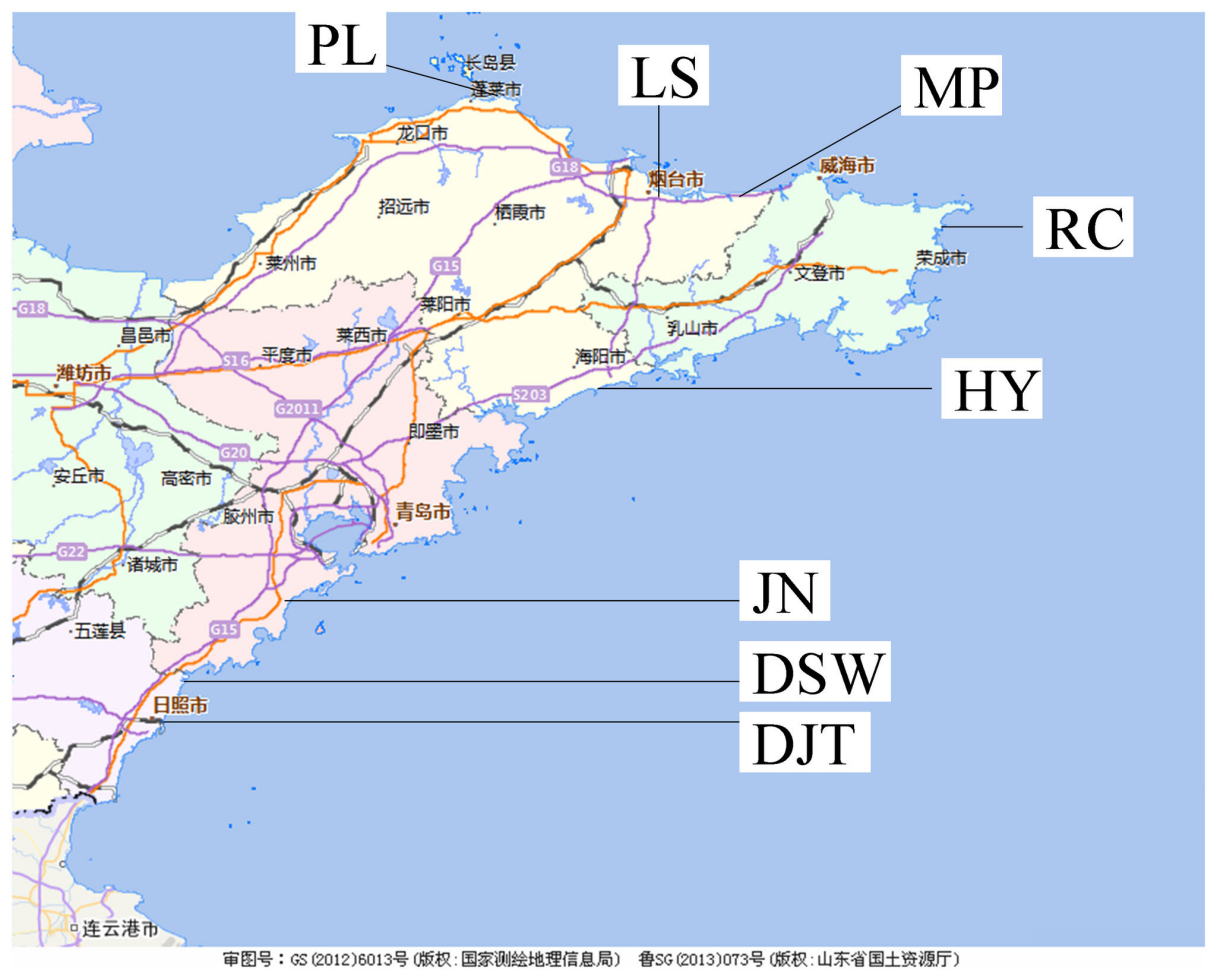
Sample coasts of the Shandong Peninsula, northern China. PL, Penglai; LS, Laishan; MP, Muping; RC, Rongcheng; HY, Haiyang; JN, Jiaonan; DSW, Dashawa; DJT, Dongjiatan.

From 2008 to 2010, we browsed sandy coasts along the coastlines in July when plants grow well, and got acquainted with all the present plants and relevant conditions. After that, we decided on eight sandy coasts for the following investigation, in view of their length adequacy and habitat typicality. The eight coasts were open for free visiting. They are located in Penglai (PL), Laishan (LS), Muping (MP), Rongcheng (RC), Haiyang (HY), Jiaonan (JN), Dashawa (DSW) and Dongjiatan (DJT) (Figure 1). Among the eight separate coasts, DSW and DJT are the nearest two, with a distance of ca. 10 km. The eight sandy coasts are separated by texture-different coasts. We observed the coasts and vegetation, and also inquired about histories of human disturbances. 

In July 2011, we further investigated coastal vegetation on supratidal zones of the coasts for comparable details. This time, we investigated each of the coasts using ten 100 m sampling lines parallel to the coastlines. The seaward edges of the coastal vegetation were designated as baselines, and ten sampling lines were 1, 2, …, 10 m away from the baselines. We recorded appearance times of every species on sample points of the lines, one sample point per meter from 0 m to 100 m (i.e., 101 sample points in total per line). We also recorded appearance times of bare spots at the sample points. Thus, we collected appearance times of all species on each coast, and the following analyses were based on these data. 

### Data analyzing

We determined geographical distributions and natural habitats of all the present species by consulting related literature [23,24]. If the distribution of a species is normally restricted to coastlines, it was considered a coastal specialist; otherwise, it was considered a terrestrial generalist, being quite common or widespread in hinterlands. The eight coasts were actually eight samples for sandy coasts. Via them, we calculated indices such as relative dominance (*RD*_*i*_), commonness (*C*_*i*_), and Levins’ niche width (*NW*_*i*_) of each species, which provided multi-information for our assessment [25]. We examined whether the *RD*_*i*_, *C*_*i*_ and *NW*_*i*_ values were different between the specialists and the generalists via T-tests [26].

$$RDi=Fi/\sum_{t=1}^{S}Ft$$

(Form. 1)

where *RD*_*i*_ is the relative dominance of the *i*th species, *F*_*i*_ is the total appearance times of the *i*th species on the eight coasts, *F*_*t*_ is the total appearance times of the *t*th species on the eight coasts, and *S* is the total number of species on the eight coasts.

Ci=Ei/n

(Form. 2)

where *C*_*i*_ is the commonness of the *i*th species on sandy coasts, *E*_*i*_ is the number of coasts where the *i*th species is present, and *n* is the total number of the investigated coasts.

$$NW_{i}=1/\sum_{j=1}^{n}{\left(Pij\right)}^{2}$$

(Form. 3)

where *NW*_*i*_ is the Levins’ niche width of the *i*th species, *P*_*ij*_ is the appearance times of the *i*th species on the *j*th coast divided by the total appearance times of the species on all the sample coasts, and *n* is the total number of sample coasts.

We analyzed the plant communities on the eight coasts using non-metric multidimensional scaling (NMS) [27]. We then disunited each community into two subcommunities of coastal specialists and terrestrial generalists. The Sørensen dissimilarities (*D*_*ik*_) of coastal specialists and terrestrial generalists, respectively, were calculated between any two of the coasts [28]. These analyses were performed via PC-ord 5.0 [29]. We further used a paired T-test to examine whether the Sørensen values of coastal specialists were significantly different from those of terrestrial generalists, which were paired if derived from the same two coasts.

$$D_{ik}=\frac{\sum_{j=1}^{S}\left|a_{ij}-a_{kj}\right|}{\sum_{j=1}^{S}a_{ij}+\sum_{j=1}^{S}a_{kj}}$$

(Form. 4)

where *D*_*ik*_ is the Sørensen dissimilarity between the coasts *i* and *k* , *a*_*ij*_ and *a*_*kj*_ are the appearance times of the species *j* on the coasts *i* and *k*, respectively, and *S* is the total number of species present at the coasts *i* and *k*.

We assessed species diversities, including richness (*S*) and Shannon–Wiener index (*H*), of all the subcommunities [30]. Richness (*S*), a simple but sound index that represents a plant community, is the number of species appearing on a coast [31]. We then calculated variation coefficients of the diversity values of the subcommunities, which are standard deviations divided by corresponding averages. Moreover, we calculated the Spearman’s correlation coefficients of ranked bareness, i.e., ratio of bare sample points to all sample points, with ranked species diversities of coastal specialists and terrestrial generalists. We also used a F-test to examine whether diversity variances among the coasts were different between the specialists and the generalists [26].

$$H=-\sum_{i=1}^{S}p_{i}\ln(p_{i})$$

(Form. 5)

where *p*_*i*_ is appearance times of the *i*th species divided by total appearance times of all species of the same subcommunity, and *S* is the number of species within the subcommunity. 

## Results

### Performances of coastal plants

The vegetation on the supratidal zones of the eight coasts comprised 11 coastal specialists: *C. kobomugi*, *C. soldanella*, *I. bartatum*, *C. pumila*, *L. mollis*, *M. sibirica*, *V. trifolia* var. *simplicifolia*, *G. littoralis*, *C. repens*, *S.* strigillosa and *L. maritimus* (Table 1). Their natural habitats are supratidal zones of sandy coasts, and we never found any wild individual of them in hinterlands of the peninsula. A total of 18 species of terrestrial generalists were found in the eight coasts, such as *C. dactylon*, *C. microiria*, *L. bicolor*, *L. davurica*, *I. cylindrica*, *O. erythrosepala*, *L. apetalum*, *C. canadensis* and *Z. japonica* (Table 1), whose distribution areas cover many hinterlands of or out of the peninsula, much broader than supratidal zones of sandy coasts. According to our observation for many years, their appearances are much more frequent in hinterlands of the peninsula than along the coastlines of the peninsula. 

**Table 1:** Components of coastal vegetation and their characteristics in the Shandong Peninsula.

Species	Family	Relative Dominance (%)	Commonness (%)	Niche Width	Desirable Habitat	Coastal Specialist
*Carex kobomugi*	Cyperaceae	24.4	87.5	5.31	SSZ	Yes
*Calystegia soldanella*	Convolvulaceae	15.7	100	6.25	SSZ	Yes
*Ischaemum bartatum*	Poaceae	13.8	87.5	2.7	SSZ	Yes
*Carex pumila*	Cyperaceae	7	100	6.01	SSZ	Yes
*Leymus mollis*	Poaceae	4.8	62.5	2.64	SSZ	Yes
*Messerschmidia sibirica*	Boraginaceae	3.4	87.5	4.31	SSZ	Yes
*Vitex trifolia* var. *simplicifolia*	Verbenaceae	3.3	75	4.17	SSZ	Yes
*Glehnia littoralis*	Umbelliferae	2	62.5	2.56	SSZ	Yes
*Chorisis repens*	Compositae	0.7	50	3.6	SSZ	Yes
*Scutellaria strigillosa*	Labiatae	0.7	25	2	SSZ	Yes
*Lathyrus maritimus*	Papilionaceae	0.7	25	1.38	SSZ	Yes
*Cynodon dactylon*	Poaceae	11.9	50	2.98	NH	No
*Cyperus microiria*	Cyperaceae	2.6	25	1.94	NH	No
*Lespedeza bicolor*	Papilionaceae	1.3	12.5	1	NH	No
*Lespedeza davurica*	Papilionaceae	0.9	12.5	1	NH	No
*Imperata cylindrica*	Poaceae	0.6	37.5	2.27	NH	No
*Oenothera erythrosepala*	Onagraceae	0.6	25	1.92	NH	No
*Lepidium apetalum*	Cruciferae	0.5	25	2	NH	No
*Conyza Canadensis*	Compositae	0.2	25	2	NH	No
*Zoysia japonica*	Poaceae	0.2	12.5	1	NH	No
*Echinochloa crusgalli*	Poaceae	0.1	12.5	1	NH	No
*Setaira viridis*	Poaceae	0.1	12.5	1	NH	No
*Spergularia marina*	Caryophyllaceae	1.8	12.5	1	Saline land	No
*Salsola collina*	Chenopodiaceae	1.7	62.5	3.92	Saline land	No
*Corispermum* sp.	Chenopodiaceae	0.2	25	2	Saline land	No
*Halogeton glomeratus*	Chenopodiaceae	0.2	25	2	Saline land	No
*Polygonum sibiricum*	Polygonaceae	0.2	12.5	1	Saline land	No
*Phragmites australis*	Poaceae	0.1	12.5	1	Wetland	No
*Astragalus adsurgens*	Papilionaceae	0.1	12.5	1	Sandy land	No

Note: SSZ, sandy supratidal zone; NH, normal hinterlands. Some species with relative dominance lower than 0.1% are omitted.

Although the species number, i.e., richness, of the specialists seemed lower, they grew considerably widespread on sandy coasts as indicated by their higher commonness (*p* < 0.001) and niche width (*p* < 0.001) than the generalists (Table 1). On average, the specialists were of higher relative dominance along coastlines than the generalists (*p* < 0.05). The three most dominant species, namely, *C. kobomugi, C. soldanella, I. bartatum*, all are coastal specialists (Table 1). 

### Vegetation dissimilarities among sandy coasts

**Figure 2:**
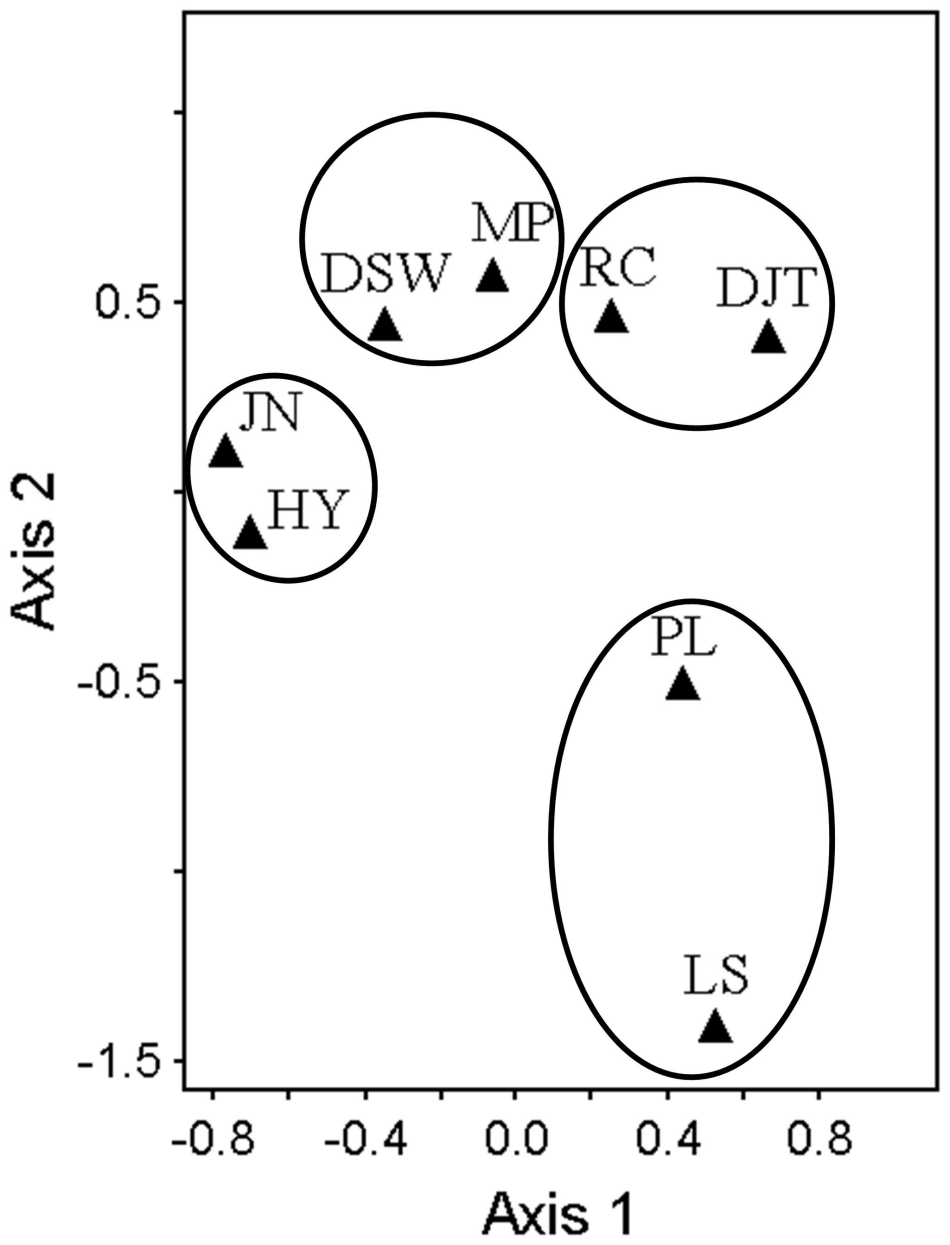
NMS ordination plot of plant communities on the eight sandy coasts.

The NMS ordination plot vividly displays the community dissimilarities among the eight coasts, which were roughly classified into four groups, associated with their histories of human disturbances, instead of geographical positions (Figure 2, Table 2). One group included JN and HY, where tourist trampling was the weakest and pond fishery was also absent. Another group included MP and DSW, where pond fishery had appeared for many years. The third group included RC and DJT, which suffered moderate trampling. The fourth group included PL and LS, where citizens and tourists often gathered for recreating or swimming. As trampling got intensified, vegetation coverage was demoted to render more bare land, and species diversities changed (Table 2). Pond fishery also affected coastal vegetation, but their effects seemed not as serious as tourist trampling (Table 2). 

**Table 2:** Conditions and vegetation diversities on the eight sandy coasts.

Sites	Disturbances	Bareness (%)	Richness		Shannon-Wiener diversity	
			Coastal specialists	Generalists	Coastal specialists	Generalists
JN	No	10	10	12	1.63	1.36
HY	Light tourism	9	9	11	1.62	1.47
MP	Pond fishery	18	8	7	1.59	1.32
DSW	Pond fishery	15	8	11	1.46	1.89
RC	Moderate tourism	35	9	6	1.78	1.50
DJT	Moderate tourism	21	8	14	1.58	0.80
PL	Heavy tourism	39	6	16	1.47	1.52
LS	Heavy tourism	41	5	4	1.43	0.61
Variation coefficients			0.21	0.41	0.07	0.32

Between any two of the coasts, Sørensen dissimilarities with respect to coastal specialists were mostly lower than those with respect to terrestrial generalists, with only three exceptions out of the 28 coast pairs (Tables 3 & 4). The paired T-test verifies the significant difference (*p* < 0.001, d.f. = 27). This indicates that vegetation dissimilarities among sandy coasts chiefly resulted from the variations of terrestrial generalists, secondarily from those of coastal specialists.

**Table 3:** Sørensen dissimilarities between coastal specialists from different sandy coasts.

	JN	RC	DJT	HY	LS	PL	DSW
RC	0.44						
DJT	0.57	0.39					
HY	0.34	0.51	0.59				
LS	0.75	0.71	0.77	0.86			
PL	0.67	0.63	0.52	0.73	0.55		
DSW	0.30	0.41	0.53	0.47	0.79	0.53	
MP	0.37	0.33	0.47	0.53	0.85	0.67	0.26

**Table 4:** Sørensen dissimilarities between terrestrial generalists from different sandy coasts.

	JN	RC	DJT	HY	LS	PL	DSW
RC	0.97						
DJT	0.96	0.99					
HY	0.90	0.93	0.98				
LS	1.00	1.00	0.25	1.00	0.25		
PL	0.93	1.00	0.56	0.92	0.37		
DSW	0.64	0.99	0.99	0.83	1.00	0.93	
MP	0.93	0.99	0.85	0.78	0.82	0.72	0.84

Note: The values surrounded by boxes are lower than their matches in Table 3; the others are not.

### Species diversity of sandy coasts

The land bareness, species richness and Shannon–Wiener diversity were affected by human activities (Table 2). Land bareness, an apparent sign of human disturbances, was negatively correlated with the diversities of coastal specialists and terrestrial generalists (all the Spearman’s correlation coefficients < -0.222, d.f. = 6), especially, significantly correlated with species richness of coastal specialists (Spearman’s correlation coefficient = -0.761, d.f. = 6, *p* < 0.05). Coastal specialists responded more deterministically to human disturbances, demonstrated by the relatively regular decrease of species richness and Shannon–Wiener diversity as human disturbances got intensified. The responses of terrestrial generalists were less deterministic because their diversities changed less regularly than the coastal specialists, with significantly larger variances to decrease or increase (F-test for richness, *p* < 0.02; F-test for Shannon-Wiener diversity, *p* < 0.01). Their variation coefficients also validated this point (Table 2). 

Pond fishery affected diversities of the coastal vegetation slightly, but tourist trampling did otherwise. Trampling could cause serious changes of species diversities, for instance, at PL and LS. 

## Discussion

### Relationships of plant species with storm surges

The specialists behaved differently from the generalists. The coastal specialists were common, widespread, and relatively dominant on the sandy coasts, which are isolated one another. The Sørensen dissimilarities of the specialists among the coasts were significantly lower than those of the generalists. These imply that the specialists may have performed easier processes of individual exchange or population turnover among separate coasts than the generalists, although other factors might play some roles [32,33]. 

**Figure 3:**
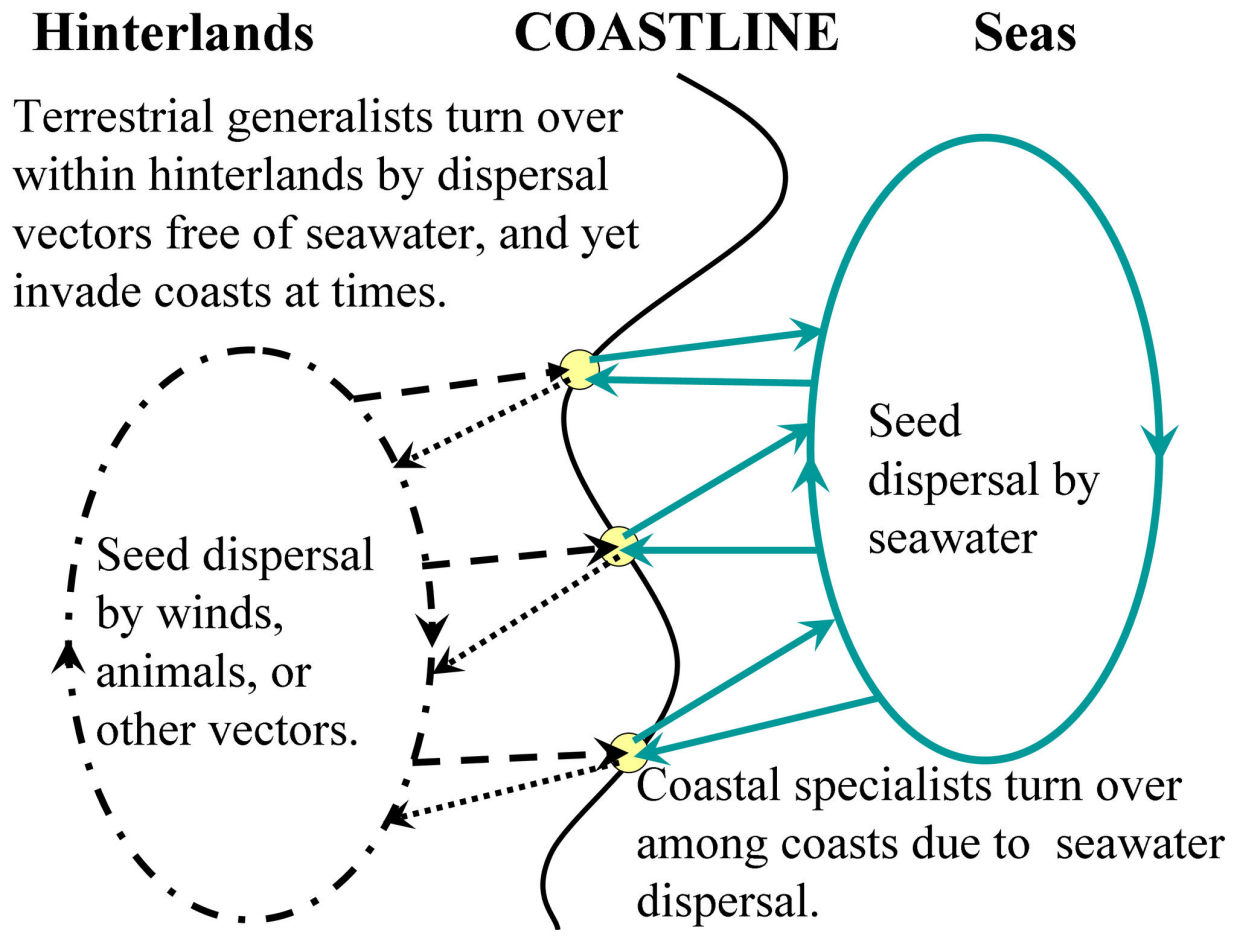
Dispersing processes of plants growing on sandy coasts.

The key to these phenomena may well be that the coastal specialists could disperse seeds easily among sandy coasts even if they are distantly isolated. Otherwise, they could not be so common and widespread on the coasts. Unlike the terrestrial generalists, they do not thrive in hinterlands of the peninsula. The broad hinterlands therefore cannot provide a passage or spot for them to continue seed dispersal. The specialists have to disperse seeds directly among similar sandy coasts, rather than depend on assistances from the hinterlands. Such dispersal is feasible in view of the available conditions of circulating seawater and their buoyant seeds, but needs facilitation of storm surges [15,16,34]. We try elucidating how their seeds are dispersed among such coasts (Figure 3). When storm surges arise, they inundate coastal specialists on supratidal zones, where they may thrive [1,20,22]. During the inundation, approximately 90% of their seeds are dispersed locally with shore currents, and approximately 10% are carried away by offshore currents into open seas [16]. Upon entering the seas, the buoyant seeds remain afloat and drift with seawater [15,16]. When storm surges arrive at other coasts, some of the seeds may land on supratidal zones. After the storm surges recede several hours later, the coasts calm and some seeds landing at favorable sites wait for potential germination and thriving. Some seeds may land on intertidal flats, but they cannot establish a local population because of frequent inundation of tides, twice a day [35]. 

Seeds of terrestrial plants are normally dispersed by winds, animals or other vectors, instead of unavailable seawater [17,36,37]. Once inundated by surging seawater, their seeds are swept off to sink to the sea bottom because they are non-buoyant, or their embryos are injured by the salty water [16,38]. Likewise, the generalists are suitable for wind or animal dispersal, and yet unsuitable for seawater dispersal (Figure 3). As storm surges flood to supratidal zones, seeds of the generalists are washed away or injured by salty water, and their vegetative bodies might be damaged as well [16,38]. Thus, their persistence is greatly inhibited by storm surges. After every storm surge, their seeds can be input again from neighboring hinterlands by winds, animals or other vectors, but fail to compete with coastal specialists for prior establishment or thriving. The generalists may be common, widespread, or even dominant somewhere in hinterlands free from storm surges, but are inferior to the specialists in occupying supratidal zones. Moreover, because the generalists cannot disperse seeds among separate sandy coasts via seawater, they inevitably take on higher Sørensen dissimilarities among the coasts. As we know, winds, animals and other vectors are not as efficient as seawater in scattering seeds along coastlines [17]. 

### Responses of plant species to human disturbances

As tourist trampling got intensified, land bareness was promoted. Meanwhile, the coastal specialists took on regular decreases in species richness and Shannon-Wiener diversities, but the generalists reacted complicatedly. Pond fishery also had effects, but it was not as detrimental as tourist trampling. The performances unveil how coastal specialists and terrestrial generalists persist on supratidal zones as human disturbances take place. 

Tourist trampling mainly takes place in summer, a good season for swimming and tourism. Summer is also good for plants to photosynthesize, grow and prepare for fruiting. Tourist trampling not only causes abnormal death of many plants, but also affects seed production and individual recruitment, thus promoting land bareness and demoting or altering species diversity. The heavier the trampling is, the greater the land bareness is and the lower the species diversities may be. Pond fishery is not as detrimental as tourist trampling, partially because pond-owners’ trampling is much less frequent. Another reason is that coastal vegetation can prevent sandy pond-walls from eroding and collapsing during storm surges, thus that pond owners like to preserve coastal vegetation as much as possible [39].

The different responses of the plants to human disturbances may also involve their characteristic seed dispersal [40]. Seeds of coastal specialists are normally adapted to seawater dispersing, but not well adapted to wind or animal dispersing because of their large, dry and colorless seeds or fruits [16]. Without seawater facilitation, seeds of the specialists cannot be dispersed as smoothly as many terrestrial plants in neighboring hinterlands. Human disturbances, especially tourist trampling, inevitably damage coastal vegetation to generate some bare spots. On such spots, many terrestrial generalists can easily invade and get established by means of convenient winds, animals, or other vectors from neighboring hinterlands, whereas coastal specialists are disabled to win the re-occupancy because their dispersal is dependant on seawater inundation, unfortunately which take place rarely with storm surges, approximately once every three years [17,41,42]. Thus, coastal specialists respond to human disturbances simply, presenting a relatively simple and deterministic decrease in species diversity. To the contrary, because terrestrial generalists can easily occupy the new bare spots, their species richness and diversities are likely to be promoted by tourist trampling and other disturbances. Even so, they can also be killed or eliminated provided the disturbances are too frequent and heavy. Given such conflicting responses, the terrestrial generalists unavoidably exhibited some irregular changes in species diversities, perhaps increasing at some times and decreasing at other times. 

## Conclusions

Generally, the coastal specialists persist well on undisturbed sandy coasts because their dispersal are facilitated by storm surges and circulating seawater. To the contrary, the terrestrial generalists are inhibited, haphazardly invading and temporarily occupying some spots of the coasts at storm-surge intervals. Human disturbances, especially unfettered tourist trampling, are adverse to the specialists, and yet act complicatedly on the generalists, perhaps being facilitative at some times and inhibitory at other times. The findings verify that coastal specialists are intrinsically superior to terrestrial generalists on supratidal zones, but human disturbances can influence the superiority of coastal specialists, by inducing vegetation degradation and diversity changes. Unexpectedly, pond fishery seems not as detrimental as tourist trampling to coastal vegetation. 
